# Magneto-Primed Triticale Seeds Studied by Micro-Raman Spectra

**DOI:** 10.3390/plants10061083

**Published:** 2021-05-27

**Authors:** Jose Alvarez, Sagrario Martinez-Ramirez, Elvira Martinez

**Affiliations:** 1 Unidad de Física y Mecánica, Departamento Ingeniería Agroforestal, School of Agricultural, Food and Biosystems Engineering, Universidad Politécnica de Madrid, Av. Puerta de Hierro, 2, 28040 Madrid, Spain; elvira.martinez@upm.es; 2 Institute for the Structure of Matter (IEM-CSIC), 28006 Madrid, Spain; sagrario.martinez@csic.es

**Keywords:** magneto-priming, micro-Raman spectroscopy, Triticale

## Abstract

The spectroscopy technique of Micro-Raman is an appropriate method to investigate the microscopic structure of internally heterogeneous (i.e., composed of multiple layers) agro-food products. The effects of applying magnetic fields (magneto-priming technique) and imbibition on the chemical makeup of Triticale seed were studied, particularly in its pericarp, germ and endosperm parts, with the help of Micro-Raman. In light of the results obtained, the magneto-primed seeds soaked in water presented a greater number of chemical compounds than the control seeds, although those treatments were not as effective as the ones with only magneto-priming. The effects of the magneto-priming treatment were especially noticeable in the endosperm due to the large number of chemical compounds identified. The seed composition differences among treatments showed that the use of Micro-Raman jointly with magneto-priming is an appropriate method to obtain and analyse information of the key components of Triticale seeds, notably regarding their pericarp and endosperm.

## 1. Introduction

### 1.1. Spectroscopy Techniques

In order to investigate the microstructural structure of different material types, different spectroscopy techniques based on the absorption, emission and scattering processes (e.g., Fourier transform infrared, nuclear magnetic resonance, hyperspectral imaging and Raman, among others) were recently developed as a non-invasive procedure of analysis, with applications in agriculture as well. These techniques showed a great deal of potential for the qualification, quantification and discovery of target chemical and physical attributes of agricultural products [1]. Among the various non-destructive analytical techniques, Raman was demonstrated to be adequate for samples that are expected to be internally heterogeneous or composed of multiple layers, such as seeds and agro-food products. Raman scattering was discovered by C.V. Raman and K.S. Krishan in 1928 and was put forward as a new form of secondary radiation. Until approximately 1986, the Raman experiments had the purpose of elucidating physical and structural aspects, shifting towards the chemical analysis afterwards.

The Raman spectra of biological materials consist of signals from all molecules present in cells, making them extremely complex. It is necessary to know the Raman patterns of the possible components of a cell to obtain the chemical information of these spectra. This technique is especially suitable for analysing biological samples [2], since water is always present on them and there is no interference with this substance. Recent developments and applications of Raman spectroscopy were reviewed in several papers, and were widely used for the detection of the chemical composition of seeds [3], seed viability evaluation (often through the starch analysis, the main component of cereal seeds) [1,3], quality evaluation of meat and fish, ingredient prediction of agricultural products [4,5,6], detection of infected seeds [6], discovery of food additives and dopants and was developed to evaluate foods, agro-products [3] and others [4].

The spectra in Raman spectroscopy result from the interactions of electromagnetic radiation with molecular vibrations in the plant or seed material [7] and we have found more uses and applications in cereal science over the last few years [8], rather than in other species. For instance, Piot et al. [9] chemically and structurally characterized the different constituents of wheat (*Triticum aestivum*) by means of Raman microspectroscopy, focusing on the endosperm (the starch granule and central and the subaleurone starchy endosperm proteins), the aleurone cell walls and the germ. Raman spectroscopy can be also used to study structural alterations resulting from different environmental conditions. Ma and Lee Phillips [8] used Raman in cereal science to learn the chemical modification degree of starches. As it will be shown later on, this is the basis of the present study made by its authors.

### 1.2. Priming Techniques

Priming techniques (osmo-priming, bio-priming, thermo-priming, halo-priming) were broadly used in numerous studies focused on enhancing seed germination parameters [9,10]. Among them, those of a physical nature (microwave, ionizing radiations, ultrasounds, magnetic fields) were also employed for improving seed germination, offering advantages over the traditional fertilizers application approach. Among these physical techniques, the magneto-priming used as pre-sowing seed treatment has many advantages, such as the low economic impact, the non-destructive testing and the environmentally friendly approach being the major ones.

The fact that the chemical make-up may be wider in some seeds than in others could be a good indicator of at which stage seeds are in the germination process: primed seeds are expected to contain a greater variety of chemical compounds. This might be a good complement to other studies that have already demonstrated the effects—visible to the human eye—of the magnetic field on seeds. Furthermore, by means of this study, the adequacy of Micro-Raman to analyse information of the key components of Triticale seeds was assessed.

The objective of this work is to study the effects of the magneto-priming technique and imbibition on the compounds identified in various parts of the Triticale seed with the help of the Micro-Raman.

## 2. Material and Methods

### 2.1. Sample Preparation

Magneto-priming was performed by placing batches of seeds into a homogeneous magnetic field with an induction of 3.72 mT (millitesla) during 10 h in an arrangement of a pair of Helmholtz coils [10]. Non-treated seeds were placed in another arrangement of Helmholtz coils during the same time but were not energized.

Seeds without magnetic treatment were labelled as A and were used as a control, magneto-primed seeds were labelled as B, batches labelled C were soaked during 5 h in 10 mL of distilled water but were not magneto-primed, and finally D-labelled treatment included both soaking and magneto-priming.

### 2.2. Micro-Raman Spectra

A Renishaw Raman Microscope System RM2000 (United Kingdom) equipped with a Leica microscope and a 633 nm He–Ne laser Renishaw RM 2000 was used. The spectra were recorded with a 25 mW laser power. The typical spectra from 100 to 4000 cm^−1^ were recorded with a resolution of 4 cm^−1^. The time acquisition was 10 s and 5 scans were recorded to improve the signal-to-noise ratio. Correct calibration of the instrument was verified by measuring the Stokes and anti-Stokes bands and checking the position of the Si band at ±520.6 cm^−1^.

Ten Triticale seeds of each treatment were cut through the demarcation line using a cutter knife and placed in the microscope of the Raman device, regardless of the treatment applied. Seeds belonging to treatments C and D were dried previously with filter paper after 5 h soaking in water. From this point on, Raman spectra were obtained from both the exterior and interior of the seeds. In the interior, six points of study were selected to analyse the germ and endosperm in each lot of seeds. An examination of the seed pericarp was also conducted in the same way.

## 3. Results

**Figure 1:**
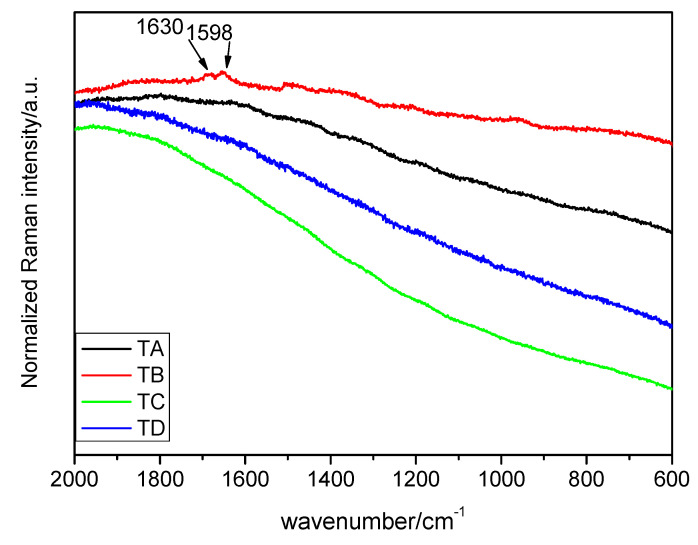
Raman spectra of the seed pericarp in the interval of 2000–600 cm^−1^ (λ = 633 nm). Treatments: TA: control, TB: magneto-primed seeds, TC: no magnetic treatment, soaked in water seeds, TD: magneto-primed and soaked in water seeds.

**Figure 2:**
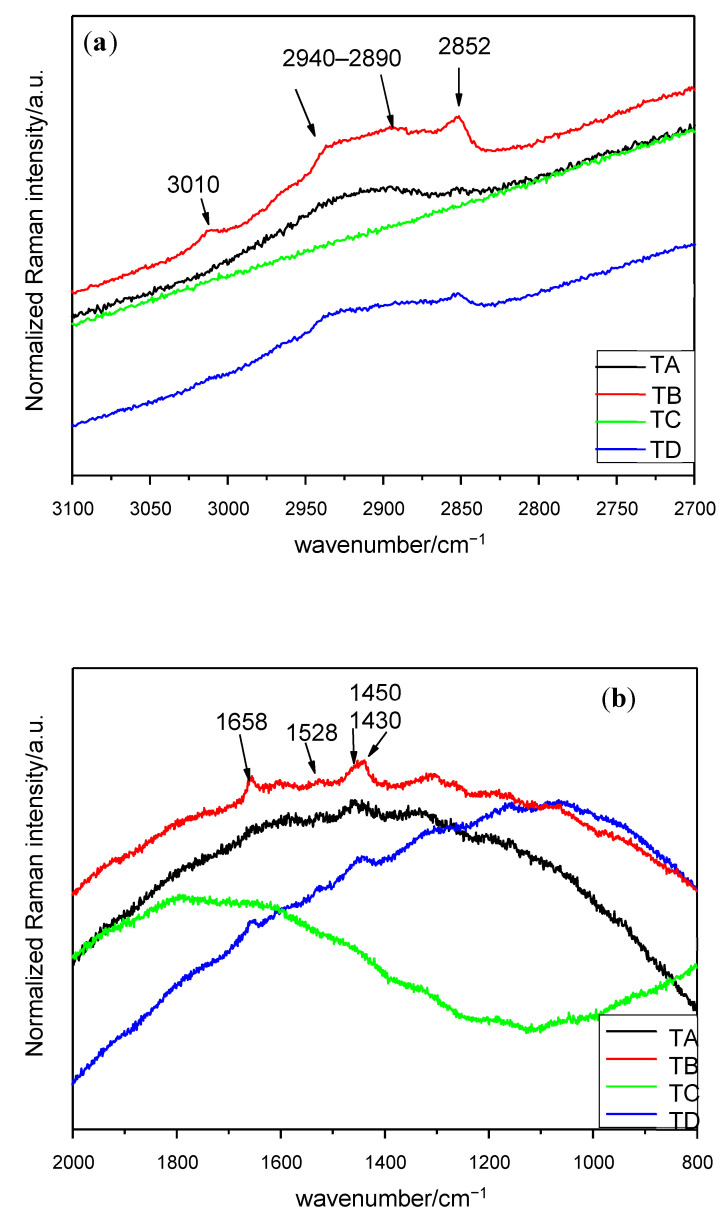
Raman spectra of seed germ in the interval (**a**) 3100–2700 cm^−1^ (λ = 633 nm), (**b**) 2000–800 cm^−1^ (λ = 633 nm). Treatments: TA: control, TB: magneto-primed seeds, TC: no magnetic treatment, soaked in water seeds, TD: magneto-primed and soaked in water seeds.

**Figure 3:**
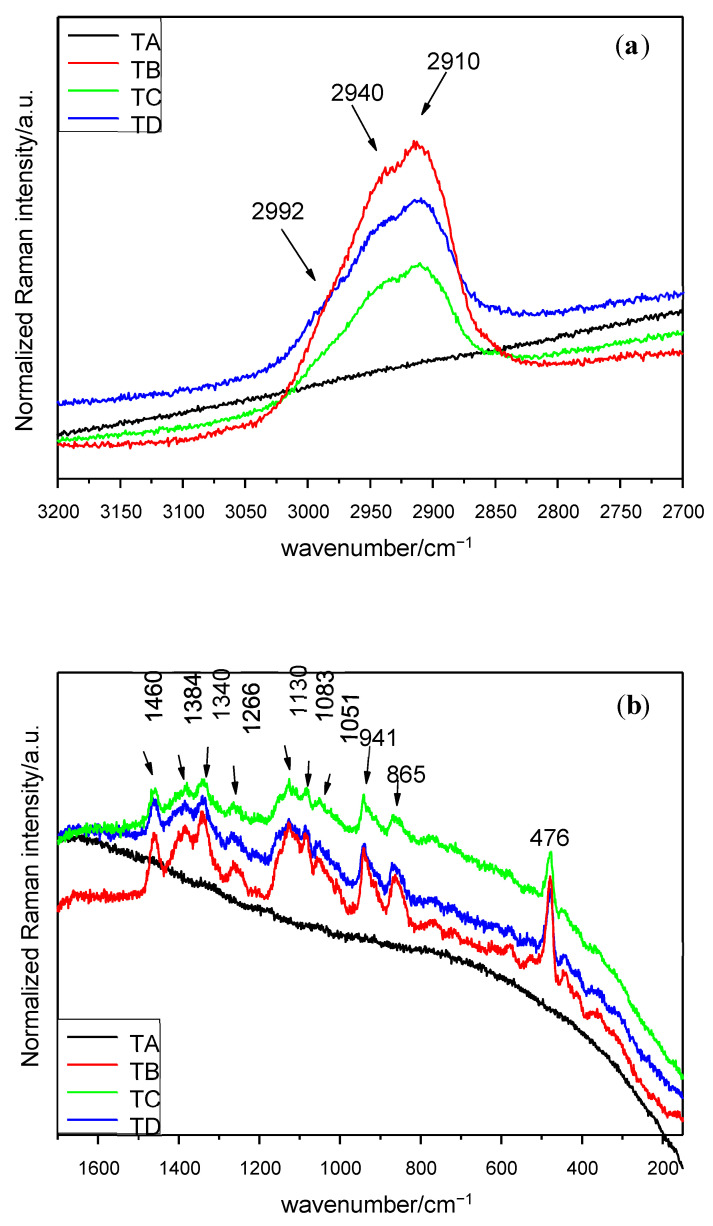
Raman spectra of seed endosperm in the interval (**a**) 3200–2700 cm^−1^ (λ = 633 nm), (**b**) 1650–150 cm^−1^ (λ = 633 nm). Treatments: TA: control, TB: magneto-primed seeds, TC: no magnetic treatment, soaked in water seeds, TD: magneto-primed and soaked in water seeds.

Having collected the Micro-Raman spectra, the different peaks obtained were identified. The Micro-Raman spectra and bands for each treatment and seed location are shown on Figure 1, Figure 2 and Figure 3 and Table 1, Table 2 and Table 3, respectively.

Figure 1 represents the Raman spectra of control and treatments of the seed pericarp. All the spectra show high fluorescence except when the sample is subjected to treatment B, in which case the part of the spectrum between 3700 and 2700 cm^−1^ reflects an odd performance with an unknown cause. However, in the interval of 2000–200 cm^−1^, several peaks can be found. The main peak is the doublet at 1630 and 1598 cm^−1^. As will be seen in the following section, the identified compounds in the pericarp are generally organic and big in size, and there may be evidence for the presence of lignin. The assignment of the signals is presented in Table 1.

The spectra concerning the germ can be seen in Figure 2. Seeds with no magnetic treatment and soaked in water (treatment C) present fluorescence and no Micro-Raman signals can be identified. Similar Micro-Raman peaks are obtained under the control and treatments B and D. It has been observed that a broad peak occurs in the interval of 2940–2890 cm^−1^ and two small bands at 3010 and 2852 cm^−1^. Additionally, four peaks have been identified in the interval of 1800–1000 cm^−1^. The assignment of the signals is presented in Table 2 and the stronger peaks are outlined in the corresponding figure.

Finally, the spectra of the seed endosperm are similar for the 3 treatments (B–D), while the control is affected by fluorescence. In the 3200–2700 cm^−1^ interval, a doublet at 2940 and 2910 cm^−1^ and a shoulder at 2992 cm^−1^ (Figure 3a) can be observed. The spectrum which displays the greatest number of peaks in the experiment is by far the interval of 1650–150 cm^−1^ (Figure 3b) from this part of the seed. The full information is included in Table 3. Within the interval 1650–150 cm^−1^, two groups of peaks appear; the first one with almost 10 units is located between 1149 and 862 cm^−1^ (this means in less than 300 cm^−1^) and the other’s peak concentration is based around 1400 cm^−1^. The most intense peak is located at 476 cm^−1^.

## 4. Discussion

In general, the most successful spectra, in terms of signals, or peaks, obtained were achieved in the endosperm part of the seed, then in the germ and finally, the seed pericarp show only a little response under this spectroscopic technique. This is in agreement with other authors who justify the poor-quality results in pericarp of several seed species due to the fluorescence phenomena that cover up Raman scattering [11,12]. Some authors go a step further and explain that fluorescence occurs because of the presence of certain substances in the pericarp [13]. Furthermore, the control sample (A) showed a lot of fluorescence in two out of the three studied parts of the seed, which means that almost no signal can be identified; with the treatments carried out on the seeds, this issue was solved.

Starting from 4000 cm^−1^ to 3000 cm^−1^, no significant change in wavenumbers or relative intensity was observed, except for the peak at 3010 cm^−1^ in the germ under treatments with magneto-priming. This can be assigned to stretching in fatty acids [14].

In many cases, some Raman bands are located in the interval of 2950–2850 cm^−1^, corresponding mostly to C–H stretching vibrations in CH_2_ and CH_3_ [15]. These molecules can be found in plenty of compounds, but the most frequent ones could be water-soluble xylan, various types of starch [5,16] or cellulose [17]. Those bands are clearer for the endosperm after all treatments.

From 1658 cm^−1^ to 910 cm^−1^ is the interval of the vibration of carbohydrates, proteins and starch. The signal at 1658 cm^−1^ may be attributed to several phenomena according to the literature (Table 2), however, the consideration of the ν(C=C) stretching vibrations of unsaturated fatty acids may be highly likely [14]. The doublet consisting of two peaks at 1630 and 1598 cm^−1^ had different assignments depending on the authors. It has been attributed to the vibration of C=C stretching in the coniferyl aldehyde unit in the lignin (1630 cm^−1^) [3], which might be consistent since this peak appears only in the pericarp. In fact, coniferyl aldehyde is also present in other hard parts of some plant species, as in the cork oak [26]. The small band at the 1598 cm^−1^ peak was assigned to the C–H stretching of the aromatic ring. Nonetheless, other authors consider these two peaks at once as the stretching vibration of phenolic ring of the 1630 cm^−1^ peak being weaker than the other [7,12,19]. In the present work, due to the high fluorescence, it is difficult to determine the real intensity of the peaks, and it is thus easy to dismiss that idea.

In the interval of 1460–1340 cm^−1^, a broad band around 1455 cm^−1^ appeared in all seed parts and can be attributed especially to CH_2_ deformation in carbohydrates [18], to fructose [19] or even to lipid content [1], depending on the authors. This part of spectra is one of the most difficult ones to analyse given the numerous chemical compounds which have Raman signal on this interval. For instance, a lot of Micro-Raman bands are related to the amylose and the amylopectin starting at this stage and up to the end of the spectra [20]. Other characteristic bands of CH_3_ or CH_2_ bending of saturated branched fatty acids or triglycerides can be observed in this region [2,12]. The signal at 1340 cm^−1^ in the study carried out in the endosperm indicates the presence of tryptophan [8,11,21,22,23].

Several bands can be found between 1266 and 1051 cm^−1^ in the treatments B, C and D in the endosperm. The bands at 1266 cm^−1^ by all mentioned treatments are related to fructose [19]. The 1130 cm^−1^ peak suggest the vibrations of C–C stretching of aliphatic chain [27] or the C–O stretching of the α 1,4 glycosidic linkages, and the presence of these is practically guaranteed because the 1460 cm^−1^ and 941 cm^−1^ peaks can be assigned to CH_2_ deformation and C–O–C stretching of such linkages, too. The 1083 cm^−1^ peak in the endosperm is very characteristic of C–O–H bending mode in some biomolecules [20]. The highest concentration of Micro-Raman bands between 1000 cm^−1^ and the end of the spectra is located at the upper end. Several peaks can be observed, mainly in the endosperm subjected to treatments B, C and D, those being generally strong. The peak at 941 cm^−1^ found in the endosperm may be related to the content of starch [11] and the one at 910 cm^−1^ to the glucopyranose ring in the same component [13]. The absorption bands in the interval of 941 and 862 cm^−1^ can be due to the C–H bending modes from the glycosidic linkage in starch [1,16]. In the region below 910 cm^−1^, the Raman shift was restricted to peaks appearing only in the endosperm spectra.

Finally, spectra acquired from treatments B, C and D showed a prominent peak at 476 cm^−1^, which can be attributed to starch [11], or more specifically, to the glucopyranose ring in starch [13].

The main supporting evidence of the effects of magnetic fields in seeds is the large number of peaks obtained under B treatment (magneto-primed seeds not soaked in water) and, to a lesser extent, under D treatment (magneto-primed seeds soaked in water) in comparison with the spectra from control seeds (treatment A), which is mainly visible in the endosperm and pericarp and suggests a greater presence of chemical compounds (a greater variety, not a larger amount). This is a fact already established by other authors [28] and is related to the ability of plant species to notice and respond more-or-less immediately to magnetic fields by altering their gene expression and phenotype [29]. The magneto-priming seems to improve the seed germination in water, although its effect is not as strong as under the treatments without imbibition. Yet it should be noted that the fact that no signals were obtained with the Micro-Raman technique does not mean that there are no certain compounds, since, due to fluorescence, it might not be possible to detect them all. What is clear is that the number of detected compounds is greater when any treatment of those mentioned is applied to the seed.

In the specific case of the germ, the bands obtained were almost similar in treatment A, treatment B and treatment D (the spectra being slightly more successful in the magneto-primed seeds). This implies that the treatments might not have any substantial effect in this part of the seed. According to several authors, seed germination in cereals has three phases culminating in the coleoptile emergence [30]. Such a phase did not take place during the experiment either because the seeds were not in contact with water, or they were only in contact with water for 5 h. This could be a possible reason for the effects of magnetism to be this insignificant in the germ.

## 5. Conclusions

For the band assignment, to the greatest extent possible, a comparison with other studies, in which either the same laser intensity was used as in the present paper or where the tested species were a cereal, was done. There are few literature sources for Micro-Raman in cereals and hardly any papers for Triticale was found. 

In this study, it was proven that the use of Micro-Raman jointly with magneto-priming is an appropriate method to obtain and analyse information of the key components of Triticale seeds, especially in the pericarp. This technique combination shows the same benefits as Micro-Raman with an imbibition treatment for seeds when analysing the endosperm.

## Figures and Tables

**Table 1:** Assignments of the Micro-Raman spectra signals of the seed pericarp under different treatments.

Pericarp				Assignments
**TA**	**TB**	**TC**	TD	[1,2,3,5,7,8,11,12,13,14,15,16,17,18,19,20,21,22,23,24,25]
	1630 m			ν(C=C) of coniferaldehyde, sinapaldehyde, stretching vibration of phenolic ring
	1598 m			ν(C-H) aromatic ring, stretching vibration of phenolic ring
	1458 w			Amylose, amylopectin, δ(CH_2_), proteins ω(C-H); δ(CH_2_) or δ(CH_3_) of saturated branched fatty acids, CH_2_ deformation in carbohydrates
	1176 w			ν(COC, CO and CC)
	935 w			Amylose, amylopectin, α 1,4 glycosidic linkage ν(C-O-C)

Signal intensity: w: weak, m: medium. Treatments: TA: control, TB: magneto-primed seeds, TC: no magnetic treatment, soaked in water seeds, TD: magneto-primed and soaked in water seeds.

**Table 2:** Assignments of the Micro-Raman spectra signals of seed germ under different treatments.

Germ				Assignments
TA	TB	TC	TD	[1,2,3,5,7,8,11,12,13,14,15,16,17,18,19,20,21,22,23,24,25]
	3010 w		3010 vw	ν asymmetric CH in fatty acids
2940 w,b	2940 w,b		2940 w,b	ν(CH)
2890 w,b	2890 w,b		2890 w,b	ν(CH)
	2852 m		2852 m	ν(CH)
	1658 m		1658 m	Amylose, amylopectin, δ(C(1)-H) and δ(C(6)-H), νs (C-O-C),ν(C=C) in unsaturated fatty acids, NH_2_ scissors in primary amines
1528 w	1528 w		1528 w	Carotenoid, zeaxanthin ν(C-C) of the polyene chain
1450 w	1450 sh		1450 sh	Fatty acids, triglycerides, δ(CH_2_) or δ(CH_3_) of saturated branched fatty acids, CH_2_ deformation in carbohydrates
1430 sh	1430 w		1430 w,b	Fatty acids, triglycerides, δ(CH_2_) or δ(CH_3_)
1160 vw	1160 vw		1160 vw	Carotenoid, zeaxanthin ν(C-C) of the polyene chain

Signal intensity: vw: very weak, w: weak, m: medium, sh: shoulder, b: broad. Treatments: TA: control, TB: magneto-primed seeds, TC: no magnetic treatment, soaked in water seeds, TD: magneto-primed and soaked in water seeds.

**Table 3:** Assignments of the Micro-Raman spectra signals of seed endosperm under different treatments.

Endosperm				Assignments
TA	TB	TC	TD	[1,2,3,5,7,8,11,12,13,14,15,16,17,18,19,20,21,22,23,24,25]
	2992 sh	2992 sh	2992 sh	C-H stretching vibrations in CH2 and CH3
	2940 m	2940 m	2940 m	C-H stretching vibrations in CH2 and CH3
	2910 s	2910 s	2910 s	C-H stretching vibrations in CH2 and CH3
	1460 m	1460 m	1460 m	Amylose, amylopectin, δ(CH_2_), proteins ω(C-H), δ(CH_2_) or δ(CH_3_) of saturated branched fatty acids, CH_2_ deformation inα 1,4 glycosidic linkage Amylose, amylopectin, δ(C-H) Amylose, amylopectin, δ(CH_2_), δ(C-H) or δ(C-O-H) Tryptophan Fructose, Unsaturated fatty acids, triglycerides, δ(-C-H), amylose, amylopectin, CH_2_OH (side chain) related mode Unsaturated fatty acids, triglycerides, δ(-C=H), amylose, amylopectin, CH_2_OH (side chain) related mode Amylose, amylopectin, δ(C-O-H), ν(C-C) of aliphatic chain, ν(C-O), α 1,4 glycosidic linkage Amylose, amylopectin, δ(C-O-H), lipids, ν(C-C), cellulose ν(C-O-C) Amylose, amylopectin Amylose, amylopectin, starch, ν(C-O-C) of α 1,4 glycosidic linkage, C-H bending Amylose, amylopectin, ν(C-O-C), glucopyranose ring, C-H bending from glycosidic linkage in starch Amylose, amylopectin, δ(C(1)-H) and δ(C(6)-H), νs(C-O-C), C-H bending from glycosidic linkage in starch Glucopyranose ring in starch
		
		
	1400 sh	1400 sh	1400 sh	
	1384 m	1384 m	1384 m	
	1340 m	1340 m	1340 m	
	1266 m	1266 m	1266 m	
		
	1240 sh	1240 sh	1240 sh	
		
	1149 sh	1149 sh	1149 sh	
		
	1130 m	1130 m	1130 m	
		
	1083 m	1083 m	1083 m	
		
	1051 m	1051 m	1051 m	
		
	941 m	941 m	941 m	
		
	928 sh	928 sh	928 sh	
		
	910 sh	910 sh	910 sh	
		
		
	865 m	865 m	865 m	
		
	862 sh	862 sh	862 sh	
		
	476 s	476 s	476 s	

Signal intensity: m: medium, s: strong, sh: shoulder. Treatments: TA: control, TB: magneto-primed seeds, TC: no magnetic treatment, soaked in water seeds, TD: magneto-primed and soaked in water seeds.

## Data Availability

The data presented in this study are available on request from the corresponding author.

## References

[1] Ambrose A., Lohumi S., Lee W.-H., Cho B.K. (2016). Comparative nondestructive measurement of corn seed viability using Fourier transform near-infrared (FT-NIR) and Raman spectroscopy. Sens. Actuators B Chem..

[2] De Gelder J., De Gussem K., Vandenabeele P., Moens L. (2007). Reference database of Raman spectra of biological molecules. J. Raman Spectrosc..

[3] Yang G., Wang Q., Liu C., Wang X., Fan S., Huang W. (2018). Rapid and visual detection of the main chemical compositions in maize seeds based on Raman hyperspectral imaging. Spectrochim. Acta Part A Mol. Biomol. Spectrosc..

[4] Li-Chan E. (1996). The applications of Raman spectroscopy in food science. Trends Food Sci. Technol..

[5] Kizil R., Irudayaraj J., Seetharaman K. (2002). Characterization of Irradiated Starches by Using FT-Raman and FTIR Spectroscopy. J. Agric. Food Chem..

[6] Lee H., Cho B.-K., Kim M.S., Lee W.-H., Tewari J., Bae H., Sohn S.-I., Chi H.-Y. (2013). Prediction of crude protein and oil content of soybeans using Raman spectroscopy. Sens. Actuators B Chem..

[7] Pelletier M.J., Pelletier C.C. (2011). Spectroscopic Theory for Chemical Imaging. Raman, Infrared, and Near-Infrared Chemical Imaging.

[8] Ma C.-Y., Phillips D.L. (2002). FT-Raman Spectroscopy and Its Applications in Cereal Science. Cereal Chem. J..

[9] Piot O., Saadi A., Autran J.-C., Manfait M. (1999). Confocal Raman microscopic characterization of the molecular species responsible for the grain cohesion of *Triticum aestivum* wheat: Effect of chemical treatment. Biomed. Appl. Raman Spectrosc..

[10] Alvarez J., Martinez E., Carbonell V., Florez M. (2019). Magnetic-time model for Triticale seeds germination. Rom. J. Phys..

[11] Piot O., Autranb J.-C., Manfait M. (2000). Spatial Distribution of Protein and Phenolic Constituents in Wheat Grain as Probed by Confocal Raman Microspectroscopy. J. Cereal Sci..

[12] Barron C., Rouau X. (2008). FTIR and Raman Signatures of Wheat Grain Peripheral Tissues. Cereal Chem. J..

[13] Jääskeläinen A.-S., Holopainen-Mantila U., Tamminen T., Vuorinen T. (2013). Endosperm and aleurone cell structure in barley and wheat as studied by optical and Raman microscopy. J. Cereal Sci..

[14] Baranski R., Baranska M., Schulz H., Simon P., Nothnagel T. (2006). Single seed Raman measurements allow taxonomical discrimination of Apiaceae accessions collected in gene banks. Biopolymers.

[15] Edwards H.G.M., Villar S.E.J., de Oliveira L.F.C., Le Hyaric M. (2005). Analytical Raman spectroscopic study of cacao seeds and their chemical extracts. Anal. Chim. Acta.

[16] Kačuráková M., Wellner N., Ebringerová A., Hromádková Z., Wilson R., Belton P. (1999). Characterisation of xylan-type polysaccharides and associated cell wall components by FT-IR and FT-Raman spectroscopies. Food Hydrocoll..

[17] Gierlinger N., Schwanninger M. (2007). The potential of Raman microscopy and Raman imaging in plant research. Spectroscopy.

[18] Cael S., Koenig J., Blackwell J. (1973). Infrared and Raman spectroscopy of carbohydrates. Carbohydr. Res..

[19] Lepore M., Portaccio M.B.E., DELLA Ventura B., Mita L., Mita D.G., Camerlingo C., Delfino I. Determination of glucose content by means of visible micro-Raman spectroscopy and interval partial least square multivariate analysis. Proceedings of the 2011 International Workshop on Biophotonics.

[20] Cael J.J., Koenig J.L., Blackwell J. (1975). Infrared and Raman spectroscopy of carbohydrates. Part VI: Normal coordinate analysis of V-amylose. Biopolymers.

[21] Parker F.S. (1983). Applications of Infrared, Raman, and Resonance Raman Spectroscopy in Biochemistry.

[22] Borchman D., Lamba O.P., Ozaki Y., Czarnecki M. (1993). Raman structural characterization of clear human lens lipid membranes. Curr. Eye Res..

[23] Grdadolnik J., Hadzi D. (1998). FT infrared and Raman investigation of saccharide-phosphatidylcholine interactions using novel structure probes. Spectrochim. Acta Part A Mol. Biomol. Spectrosc..

[24] Schulz H., Baranska M., Baranski R. (2005). Potential of NIR-FT-Raman spectroscopy in natural carotenoid analysis. Biopolymers.

[25] Tu A.T., Lee J., Milanovich F.P. (1979). Laser-Raman spectroscopic study of cyclohexaamylose and related compounds, spectral analysis and structural implications. Carbohydr. Res..

[26] Conde E., Cadahía E., García-Vallejo M.C., De Simón B.F. (1998). Polyphenolic Composition ofQuercus suberCork from Different Spanish Provenances. J. Agric. Food Chem..

[27] Piot O., Autran J.-C., Manfait M. (2001). Investigation by Confocal Raman Microspectroscopy of the Molecular Factors Responsible for Grain Cohesion in the *Triticum aestivum* Bread Wheat. Role of the Cell Walls in the Starchy Endosperm. J. Cereal Sci..

[28] Araújo S.D.S., Epaparella S., Edondi D., Ebentivoglio A., Ecarbonera D., Balestrazzi A., Epaparella S., Edondi D., Ebentivoglio A., Ecarbonera D. (2016). Physical Methods for Seed Invigoration: Advantages and Challenges in Seed Technology. Front. Plant Sci..

[29] Sarraf M., Kataria S., Taimourya H., Santos L.O., Menegatti R.D., Jain M., Ihtisham M., Liu S. (2020). Magnetic Field (MF) Applications in Plants: An Overview. Plants.

[30] Pérez García F., Martínez-Laborde J.B. (1994). Introducción a la Fisiología Vegetal.

